# Characteristics of New England India Pale Ale Beer Produced with the Use of Norwegian KVEIK Yeast

**DOI:** 10.3390/molecules27072291

**Published:** 2022-03-31

**Authors:** Joanna Kawa-Rygielska, Kinga Adamenko, Witold Pietrzak, Justyna Paszkot, Adam Głowacki, Alan Gasiński

**Affiliations:** Department of Fermentation and Cereals Technology, Wrocław University of Environmental and Life Sciences, Chełmońskiego 37, 51-630 Wrocław, Poland; kinga.adamenko@upwr.edu.pl (K.A.); witold.pietrzak@upwr.edu.pl (W.P.); justyna.paszkot@upwr.edu.pl (J.P.); adam.glowacki@upwr.edu.pl (A.G.); alan.gasinski@upwr.edu.pl (A.G.)

**Keywords:** beer, brewing, unconventional microorganisms, KVEIK yeast, fermentation, GC/MS, HPLC

## Abstract

The aim of this research was to determine the potential of four unconventional Norwegian yeasts of the KVEIK type to produce NEIPA beer. The influence of yeast strains on fermentation process, physicochemical properties, antioxidant potential, volatile compounds, and sensory properties was investigated. The KVEIK-fermented beer did not differ in terms of physicochemical parameters from the beer produced with the commercial variants of US-05 yeast. The yeast strain influenced the sensory quality (taste and aroma) of the beers, with KVEIK-fermented beer rating significantly higher. The antioxidant activity of the tested beers also significantly depended on the yeast strain applied. The beers fermented with KVEIK had a significantly higher antioxidant potential (ABTS^•+^) than those fermented with US-05. The strongest antioxidant activity was found in the beer brewed with the Lida KVEIK yeast. The use of KVEIK to produce NEIPA beer allowed enrichment of the finished products with volatile compounds isobutanol, 2-pentanol, 3-methylobutanol, ethyl octanoate, and ethyl decanoate.

## 1. Introduction

Over the centuries, human activity has led to the differentiation of *Saccharomyces cerevisiae* yeast strains. One of the areas where this phenomenon was particularly evident was the fermentation industry, which was dominated by brewing. Regardless of the region of origin, brewer’s yeast was maintained by using it constantly and moving it from one vat to another, as well as dividing the yeast between adjacent breweries in the event of its spoilage [1]. However, with the increasing industrialization of Europe coupled with the convenient availability of commercial yeast and pure cultures, the traditional yeast obtained through centuries of selection has lost its importance. Despite this, the recent beer revolution has led to the establishment of a large number of small and medium-sized breweries pursuing new unique flavors and aromas that are not provided by pure cultures obtained in laboratories, e.g., 2-pentanol, ethyl octanoate, ethyl decanoate, and furfural [2]. Thus, there has been a lot of interest in using unconventional yeasts, such as KVEIK yeast.

The use of KVEIK yeast in the production of specialty beers could be very interesting from the beer marketing point of view. As reported by Nielsen.com, the segment of specialty beer accounted for 2.7% of the whole Polish beer sector sales in 2017, with a sales increase of 16.9% in comparison to the previous year, making “beer specialties” the second-highest-growing beer segment after non-alcoholic beers [3]. This suggests that harnessing KVEIK yeast to produce distinct beers with interesting sensory features might be economically viable for market introduction due to the growth of the segment of unconventional beer consumers.

KVEIK yeasts have long been known in Norway and other parts of Scandinavia. Initially, they were used to both make bread and produce beer. Only the centuries-old selection of home brewers, mainly Norwegian ones, who kept the traditional methods of brewing beer, as well as the yeast strains used in the process, led to their separation from beer, brewery, or environment. The area is famous for the traditional brewed stjørdalsøl, konnjøl, and maltøl. KVEIK yeasts are divided into two separate subgroups within their group. The first includes yeasts from the Granvin, Stranda, Laedal, and Voss regions, whereas the second—yeasts from the Sykkylven, Hornindal, and Stordal regions. Interestingly, both groups correspond geographically to the north and south of the Jostedal glacier. The results of the latest genotyping were reported in 2018 [4]. KVEIK yeast strains from nine different Norwegian sources were characterized via PCR fingerprinting, whole-genome sequencing of selected strains, and phenotypic screens. The authors found that the studied KVEIK strains were genetically distinct from other domesticated *Saccharomyces* strains [4].

KVEIK yeast is stored in a very specific way, namely, on special wooden structures in the dried state. These wooden structures are incised logs or special oval-shaped structures. Such a wooden structure is immersed in the yeast slurry formed after fermentation, then in flour or ash, and finally dried, so that it can be stored for long periods, lasting a year or more, while the liquid slurry would deteriorate very quickly. Interestingly, some of these have different composition and properties. KVEIK can therefore represent a contrasting example of yeast constructions can be dated at least to 1621, suggesting that the KVEIK yeast thicket was used long before that date. Such a method of storing the slurry means that, in addition to brewer’s yeast, airborne yeasts and bacteria can also be found on these constructions. Therefore, depending on the region, they may substantially differ in terms of those that have been domesticated and kept in geographically isolated brewing traditions, independent, and parallel to the production of industrial beer [5].

Due to the small number of studies on KVEIK yeast, its characteristics have not been fully understood yet. In addition, the traditional yeast thicket obtained for research consists of various strains very often featuring completely different properties, which makes both the research and inference difficult. Often, the action of several strains together with the bacteria produces completely different results than with a single strain [1]. However, most KVEIK strains differ phenotypically from the commonly known and used ale yeasts (top fermenting yeasts). The distinguishing features of KVEIK yeast are strong flocculation, high fermentation rate, no phenolic aftertaste, exceptional resistance to temperature during fermentation (>30 °C), no or small amounts of 4-vinylguaiacol produced (responsible for the aroma of cloves), and high alcohol tolerance, as well as a unique flavor profile. The development of such features could have been affected by dry storage on the aforementioned wooden structures [6,7,8,9].

Due to their characteristics, the KVEIK yeast strains may prove extensively useful as a new group of brewer’s yeast. The selected strains allow the preservation of the appropriate physicochemical characteristics of New England Pale Ale style beers, which are also characterized by antioxidant activity as well as a new and interesting profile of volatile compounds and sensory properties. To the best of our knowledge, there is only one work in the available literature addressing the use of KVEIK yeasts for brewing stout-style beer, while it is very important to control the brewing of light beers when using them. In stout-style dark malts, the Maillard reaction compounds affect the metabolism of *Saccharomyces cerevisiae* yeast. Their addition in the technological process affects both the basic technological parameters of beers as well as the profile of compounds that are byproducts of ethanol fermentation and influence the sensory quality [10]. There are certain differences in the production technology of dark and light beers which can directly affect the beer production process with the use of KVEIK yeast. That is why we have decided to identify the possibility of harnessing KVEIK yeast in the production technology of light beer with an example of the NEIPA style.

## 2. Results and Discussion

### 2.1. Basic Physicochemical Parameters

Table 1 presents basic physicochemical parameters of the tested beers. Beer samples were analyzed for ethyl alcohol concentration, apparent and real extract content, density, apparent and real fermentation degree, energy value, and pH. Determinations were carried out at three production stages: after primary fermentation, after secondary fermentation, and after beer aging in bottles. After the primary fermentation, the highest ethyl alcohol concentration (3.97% *v*/*v*) and the highest fermentation degree (% of sugars converted to alcohol) (58.98%) were analyzed in the US-05 sample. At this stage of fermentation, the worts fermented with KVEIK yeast had a similar alcohol concentration, which reached 3.60% *v*/*v* and was higher by 0.37 percentage points (p.p.) compared to that determined in the US-05 wort. At this stage, the fermentation degree of KVEIK yeast was lower by 1.21 p.p. Preiss et al. observed a higher fermentation dynamics (as measured by weight of CO_2_ liberated in the first three days of the process) for most of the KVEIK strains tested compared to the control yeast; however, they fermented wort at a higher temperature, i.e., at 30 °C. KVEIK yeasts are capable of beer fermentation at high temperatures [4]. This difference may also be due to the carbohydrate profile of wort used in the cited study. After secondary fermentation, ethyl alcohol concentration increased in all samples to a similar level of 4.71% *v*/*v*, on average. At this fermentation stage, pH was observed to decrease, with the greatest drop noted in the LK sample (pH = 4.28). In turn, the pH value of the wort fermented with the industrial yeast strain was pH = 4.56.

At the stage of beer aging in bottles, ethyl alcohol concentration increased in all beer types to the mean value of 5.3% *v*/*v*. The fermentation degree noted in all samples fermented with KVEIK yeast and US-05 yeast reached 70.51% and did not differ significantly among individual samples. Preiss et al., who compared ethyl alcohol concentration and fermentation degree of worts fermented with various KVEIK yeast strains and industrial strains, also found no statistically significant differences between them [4]. Although this lack of significant differences suggests that KVEIK yeast shares similar technological features with strains commonly used in brewing, it may be important from the technological point of view. During beer aging in bottles, the pH value increased in all samples to pH = 4.67 on average. There were no significant differences in its values among the beer variants. The KVEIK-fermented beers had a lower energy value than conventional ale beers. The lowest energy value was determined for VK beers (34.04 kcal/100 mL) and the highest one for US-05 beers (38.03 kcal/100 mL). This difference was probably due to a negligibly higher alcohol content of the US-05 sample.

The analysis of the basic physicochemical parameters of beers fermented with KVEIK yeast demonstrated that they had similar technological features to those fermented with commercial yeast strains, which was indicated by insignificant differences in their ethyl alcohol concentration, fermentation degree, and pH value.

### 2.2. HPLC Analysis

Table 2 presents the concentrations of starch hydrolysis products (glucose, maltose, maltotriose, and dextrins (DP4+)) and glycerol in the wort at different fermentation stages. Acetic and lactic acids were not detected at any of the fermentation stages, which may indicate no bacterial contamination of beer or the incapability of the yeast strains to produce these acids under experimental conditions. All tested yeast strains converted the available maltose during primary fermentation. A small amount of glucose was determined after primary fermentation in the worts fermented with the control strain (US-05) and LK, with a significantly higher content of residual glucose found in the case of the latter strain. After secondary fermentation, trace amounts of glucose (below 0.5 g/L) were detected only in the beer produced with the US-05 strain. The KVEIK group yeast proved better in maltotriose fermentation than the control strain. After the primary fermentation, maltotriose was detected in the worts fermented with two KVEIK strains (HVK and FM53 which fermented ca. 91.3 and 89.9% of maltotriose, respectively) and US-05 strain (85.2%). Maltotriose was not detected in any of the beers tested in the successive stages of the experiment. Significant strain-dependent differences were also observed in the consumption of dextrins at various beer production stages. After primary fermentation, almost all tested strains fermented the same amount of dextrins (2.18 g/L, 6.9% on average), whereas the VK2 strain fermented ca. 0.5 g dextrins/L (ca. 1.6%). Young beers (after silent fermentation) differed significantly in a dextrin fermentation degree. At this stage of the fermentation process, the LK strain consumed almost 50% of dextrins present initially in the wort, whereas the other KVEIK strains fermented ca. 15.7% (VK2) and ca. 8.5% (FM53) of oligosaccharides. At the same time, the control strain (US-05) consumed ca. 40.8% of dextrins. In the ready beers (after aging), the VK2 and FM53 strains fermented more dextrins than in the previous production stage (40.7 and 47.2% of the initial concentration, respectively). The HVK strain was the least potent in fermenting oligosaccharides (it fermented 24% of dextrins, i.e., only 12.7% more than in the previous fermentation stage). The LK and US-05 strains showed the highest capability for dextrin fermentation from the wort (55.4 and 57.1%, respectively); however, the control strain statistically fermented the highest amount of oligosaccharides. All KVEIK strains produced more glycerol than the control strain, with the FM53 and VK2 strains being the best producers. Glycerol concentration decreased after secondary fermentation in most beer types (especially in those produced with the LK strain, where glycerol concentration decreased to ca. 31% compared to the previous fermentation stage). Glycerol consumption from beer after the primary fermentation could, presumably, be due to slight oxygenation of the fermentation medium during beer decanting from the yeast sediment, which made the yeast capable of utilizing glycerol in the absence of easily-digestible carbon sources, i.e., sugars. In ready beers, the highest glycerol concentrations were determined in the samples fermented with FM53 and VK2 strains; however, the final glycerol concentration in the beer produced with VK2 strain was similar to that in the beer produced with the LK strain. In turn, glycerol concentrations in the beers fermented with the control and HVK strains were statistically similar.

Preiss et al. analyzed a total of nine samples of KVEIK yeast obtained from Norwegian beer producers [4]. They found that most of the samples consisted of 1–9 *S. cerevisiae* strains while 25 different isolates were found in one sample. The isolates found in the KVEIK samples studied by them differed in technological features, such as the dynamics of fermentation, ethyl alcohol production, or maltotriose consumption, which suggests that the unique technological features of KVEIK yeast are due to the dynamics of the yeast population and their interactions during fermentation. This study demonstrated that two of the tested KVEIK strains (LK and VK2) were characterized by a much faster maltotriose fermentation (after the primary fermentation), and that two other strains (FM53 and VK) showed only slightly (although statistically significant) accelerated maltotriose fermentation compared to the control strain. The above finding confirms significant differences in the technological features of KVEIK strains observed by Preiss et al., where the maltotriose fermentation degree ranged from 0 to almost 100% after a 12-day fermentation of the wort with an extract content of 12.5 °Plato at 30 °C, with more than half of the KVEIK yeast isolates tested ensuring a high (over 80%) level of this sugar fermentation [4]. Krogerus et al. (2018) performed a detailed analysis of 34 technological features of the KVEIK yeast isolate called Muri [11]. By examining its genotype, they found it to be an atypical, natural hybrid of *S. cerevisiae* and *S. uvarum* yeasts (i.e., top- and bottom-fermenting strains, respectively) sharing the features of both parent species. In addition, they demonstrated that the technological features of the Muri isolate were similar to those of the top-fermenting yeast, including, e.g., the ability to utilize dextrins, which was also observed in this study. The increased glycerol production by the studied KVEIK strains may be related to their high, optimal fermentation temperature (>30 °C), which is one of the factors positively influencing glycerol production by yeasts [12]. To sum up, the HPLC analyses of the samples performed in this study and in other investigations reported in the available literature fail to clearly indicate the technological features of KVEIK yeast isolates due to the significant differences in their genotypic and phenotypic characteristics. It should also be taken into account that traditional methods of KVEIK yeast preservation between fermentation stages may result in infections and a change in the population dynamics of various strains, which may in turn lead to further changes in the characteristics of these yeasts.

Under the applied experimental conditions, the KVEIK yeast did not differ significantly from the control, commercial yeast strain US-05, in terms of parameters of both the fermentation process and the technological process. Considering the thermal stability of KVEIK yeast strains and their ability to carry out metabolic processes under unfavorable conditions, it can be concluded that they have a high potential in the brewing technology.

In addition, they were characterized by a similar ability to utilize maltose and glucose as the investigated industrial strain and a better ability to utilize maltotriose. They also showed different ability to utilize dextrins present in the wort.

### 2.3. Analysis of Polyphenolic Compounds

The antioxidative activity of beer is mainly ascribed to the phenolic compounds of malt and hop and to melanoidins produced during heat treatment of malt, mash, and brewing wort [13], as well as to additives, mainly fruits [14,15,16]. The content of phenolic compounds affects the key quality attributes of beer, i.e., clarity, color, and taste, as well as its storage stability [17]. The transformation of these compounds during ethanolic fermentation with brewer’s yeast is of particular interest. Worts and beers after primary fermentation, secondary fermentation, and aging were determined for the total phenolics content (TPC) and antioxidative activity, and respective results are presented in Table 3.

The total content of phenolic compounds in the wort was at 160.1 mg GAE/L. In beers, it reached 219.7–334.0 mg GAE/L after primary fermentation and 253.1–354.3 mg GAE/L after secondary fermentation. At both stages of fermentation, the highest TPC was determined in the control sample (US-05). Among the KVEIK-fermented beers, the highest TPC after the primary fermentation was determined in the LK and VK2 samples (320.0 and 306.7 mg GAE/L, respectively). After the consecutive stage of fermentation, the VK2 sample had also the second-highest TPC, after the control US-05 sample (295.2 mg GAE/L). The content of phenolic compounds of beers changes over the technological process. Its increase is observed during wort hopping, main fermentation, and post-fermentation [13].

A TPC decrease after secondary fermentation, compared to the primary fermentation, was demonstrated for all beer variants, regardless of the yeast strain used to produce them. After aging, the TPC of the finished beers ranged from 179.7 to 332.6 mg GAE/L. Its highest value was determined in the FM53 beer and was 12.2% higher than in the control sample. The TPC determined in the other beers was lower compared to the control sample. The HVK and VK2 beers did not differ statistically significantly in the content of polyphenolic compounds, showing TPC values of 275.3 and 273.9 mg GAE/L, respectively. In turn, the LK bear had the lowest TPC (179.7 mg GAE/L) among the finished beers and all other analyzed beers.

A decreased concentration of phenolics after aging confirms results of earlier studies [13]. In contrast, the activity of yeast esterase of ferulic acid leads to the release of phenolic acids during primary and secondary fermentation, and aging, which may result in their increased content [17]. In addition, during fermentation, phenolic compounds enter into reactions with proteins and polysaccharides of yeast cells. They are adsorbed to their cell walls, and together with the yeast sludge, fall down onto the bottom of a fermentation tank as a result of flocculation, and thus become separated from beer [18]. This phenomenon could underlie TPC decrease, in particular beer fermentation stages, especially considering the fact that the KVEIK yeast strains used in the present study exhibit a high flocculation capability [4].

The antioxidative activity of worts and beers was analyzed with three methods (DPPH^•^, ABTS^•+^, and FRAP). The values determined for various beer variants varied in particular fermentation stages depending on the yeast strain applied. After primary fermentation, the highest antioxidative potential was determined for LK (DPPH^•^), FM53 (ABTS^•+^), and VK2 (FRAP) beers. This, however, changed after secondary fermentation and aging. Among the finished beers, the LK beer showed the highest DPPH^•^ and ABTS^•+^ scavenging capability (2.00 and 7.21 mM TE/L, respectively). The use of KVEIK yeast enabled increasing the antioxidative potential of all beers (analyzed with the ABTS^•+^ assay) compared to the control US-05 sample but contributed to decreased FRAP values. The highest FRAP value was determined for the control beer (1.33 mM TE/L). In turn, FM53 beer showed the highest ferric-reducing ability among the KVEIK-fermented beers (1.14 mM TE/L).

The KVEIK yeast strains are incapable of decarboxylating hydrocynnamic acids, which represent one of the groups of beer phenolics, including, e.g., 4-vinylguaijacol. In terms of this capability, yeasts are classified to POF+ or POF− groups, namely, to the groups of yeast capable or incapable of producing the so-called phenolic off-flavor, which adversely affects the sensory profile of beer [19]. Due to mutations in PAD1 and FDC1 genes, the KVEIK yeast strains are incapable of producing phenolic acids of 4-vinylguaiacol, being a volatile compound synthesized, among other things, during transformations induced by brewing yeast strains used to manufacture wheat beers, *Saccharomyces diastaticus* yeast, and many other wild and top-fermenting yeast strains. Because 4-vinyl derivatives are weaker antioxidants than their precursors (phenolic acids), these transformations could diminish the antioxidative activity of beers after fermentation [20]. The higher antioxidative activity compared with the control was noted for LK beer in the DPPH^•^ assay, and for HVK, LK, and WM53 variants in the ABTS^•+^ assay.

The use of KVEIK yeast strains in ethanolic fermentation enabled increasing the antioxidative potential of all beers (analyzed with the ABTS^•+^ assay) compared to the control US-05 sample but contributed to decreased FRAP values. The ethanolic fermentation led to an increase in the antioxidative activity. Changes in the antioxidative potential depended on the yeast strain applied. Among the finished beers, the one fermented with Lida KVEIK yeast showed the highest DPPH^•^ and ABTS^•+^ scavenging capability. The content of phenolic compounds in beers also depended on the yeast strain used, with the highest content obtained in the beer fermented using KVEIK FM53 yeast.

### 2.4. Analysis of Volatile Compounds

The GC-FID with external standards allowed us to identify and quantify 21 volatile compounds, the predominating among which was a group of alcohols with as many as nine identified compounds (Table 4).

The other groups included esters (five compounds), aldehydes (four compounds), and pyrazines (three compounds). All beers had a similar total content of volatile compounds, ranging from 390.842 mg/L for VK2 beer to 400.846 mg/L for FM-53 beer. The control beer (US-05) had a slightly lower content of these compounds (395.737 mg/L) compared to the mean content of volatiles determined for all analyzed beers (396.521 mg/L). In addition, it had the highest content of ethyl acetate among all beers tested, which reached 120.86 mg/L and was almost 20% higher compared to FM-53, VK2, and HVK samples (95.95–96.72 mg/L). The content of ethyl acetate determined in the LK beer (114.54 mg/L) was significantly higher than in the other beers produced with KVEIK yeast, but still lower than in the US-05 beer. However, it is worth emphasizing that the concentration of ethyl acetate, featuring a fruit flavor, was 3–4 times lower in all beers tested compared to the bottom-fermentation beers [21]. A study conducted by Preiss et al. with 25 strains of KVEIK yeast demonstrated that part of them were able to produce ethyl hexanoate, octanoate, and decanoate in beer wort in concentrations exceeding the detection level and amounting for 0.21 mg/L, 0.9 mg/L, and 0.20 mg/L, respectively [22]. Among the aforementioned esters, only ethyl hexanoate was detected in the control US-05 sample in the higher amount (0.38 mg/L) than in some beers produced with KVEIK yeast (0.30–0.35 mg/L in HVK, LK, and VK 2 samples), whereas its content was the highest in the FM53 beer (0.44 mg/L). The content of ethyl decanoate was above the detection limit in all beers. However, its concentration in the beers fermented with KVEIK yeast was 220% to 360% higher than in US-05 beer (3.31 mg/L) and ranged from 7.26 mg/L in VK2 beer to 12.16 mg/L in LK beer. The same was observed for ethyl octanoate, the concentration of which was higher in all KVEIK-fermented beer samples fermented (0.90–1.01 mg/L) than in the control US-05 beer (0.80 mg/L). Worthy of notice is that only in the US-05 was its concentration below the detection limit, reaching 0.90 mg/L. Another important group of compounds occurring in fermented beverages is the so-called higher alcohols that may impart fruity notes (banana, orange, mango, pineapple) to the flavor bouquet [23]. The key representatives of this group include phenylethyl alcohol, 2-methylbutanol, and 3-methylbutanol. The VK2 sample had the lowest contents of these alcohols, but in turn had the highest contents of 1-propanol (70.22 mg/L) and isobutanol (56.97 mg/L). The highest contents of 2-methylbutanol and 3-methylbutanol were determined in the HVK beer (35.38 mg/L and 124.70 mg/L, respectively). In turn, the highest concentration of phenylethyl alcohol was determined in the FM-53 sample (55.68 mg/L) and it was 150% higher than in the US-05 beer. The HVK beer was the only beer where no 2-butanol was detected, whereas FM53 and LK samples had detectable amounts of 2-pentanol (0.14 mg/L and 0.49 mg/L, respectively). An interesting feature of the composition of volatile compounds in the analyzed beers was the content of acetaldehyde, a toxic compound which, when present in too-high concentrations, imparts the beverages a solvent-like and overwhelming aroma [24]. In the LK and VK2 samples, its concentration was 125–130% higher than in the control US-05 beer (4.05 mg/L), whereas in the HVK beer it was significantly lower (1.83 mg/L). Noteworthy is also the absence of 2,3-diethylpyrazine in the FM-53 sample, and its similar concentration, amounting to 0.42–0.45 mg/L, in the other samples. Beers prepared with KVEIK yeast were also characterized by a reduced content of furfural, which is a toxic compound for microorganisms (from 5.65 mg/L in VK2 to 8.47 mg/L in HVK), compared to US-05 (9.19 mg/L). This may indicate that KVEIK yeast strains have an improved ability to metabolize this compound [12].

The analyzed KVEIK yeast produced a smaller amount of ethyl acetate and a larger amount of ethyl decanoate compared to the industrial yeast strain. Moreover, the determined content of ethyl decanoate exceeded the detection level.

### 2.5. Sensory Analysis

The beers were assessed for foaminess, clarity, color, saturation, bitterness, aroma, and taste. All beers produced with KVEIK yeast received the same scores as the beer fermented with a commercial yeast strain US-05 (Table 5).

In addition, the sensory analysis showed no significant differences among the NEIPA beers produced with various KVEIK strains. The highest number of points was given by the panelists to the FM53 beer, which was, by nearly 13 points more than the score given to the control beer (US-05), rated the highest for its taste (by as much as 8 points). The lowest number of points was given to the VK2 beer, but still it was 2 points more compared to the US-05 beer. In the descriptive assessment of beers, consumers indicated VK2 beer as non-alcoholic and characterized by an uncommon and unusual aroma compared to classic beer styles. Noteworthy, the consumers indicated that the US-05 beer had a very poorly perceptible aroma compared to the other evaluated beers and that it most closely resembled the commercial beers. This is a correct observation because this beer variant was produced using a common and commercial yeast strain. In the case of all beers, the panelist described the aromas of malt as those resembling grain, bread, straw, and hay, whereas the aromas of hops were described as citrus, herbal, tea, and resinous. The indicated ester aromas were banana, pineapple, and apricot. Among other aromas, consumers also mentioned fruity and wine ones. The quality of the bitterness was described as delicate and its finish as short. According to the panelists, the taste was dominated by citrus, herbal, flower, and resin notes. There are no studies available in the literature regarding the influence of KVEIK yeast on the development of the sensory properties of beer. Only the review article by Bråtå (2017) mentioned that KVEIK yeast directly affected the fermentation process and, thus, the finished product through its additional aromatization [25]. Until now, *Saccharomyces cerevisiae* Hordinial KVEIK yeast has been used only to produce fermented sour whey beverages featuring a mild sensory profile. The aromas of citrus fruits and apples that could be produced by this yeast strain during fermentation were moderately perceptible [26]. The amount and type of esters produced by brewer’s yeast during the alcoholic fermentation depend on the expression of genes and the production of enzymes responsible for the formation of individual compounds. This, in turn, depends not only on the yeast strains, which differ genotypically, but also on the stage of fermentation, which is a dynamic process [27].

As shown in this study, the fermentation performance of KVEIK was not clearly explained, which was also reported by the previous studies [2,4,11]. One way to point out the fermentation characteristics of KVEIK yeast when provided in mixed culture is to evaluate its monocultures in the ranges of sugars consumption, ethanol, and volatiles production along with the sensory features of final beers. This approach would, however, not provide an exact insight into the fermentation course of the KVEIK yeast available on the market due to (1) the dynamics of yeast population during beer fermentation when one strain might outcompete other strains present in the inoculum, and (2) the yeast–yeast interactions during fermentation which may result in variations of process course and product features [18]. Therefore, showing the fermentation potential and beverage quality of beers fermented by each individual yeast strain potentially present in commercially available KVEIK yeast starters might not be relevant from the practical point of view for the commercial beer production practice where the evaluation of the whole inoculum performance in a particular environment will be of higher value.

The KVEIK yeast strains can be successfully used to produce not only traditional Norwegian beers but also new-wave beers, which has been proven in the example of the New England India Pale Ale style. Their use does not interfere with the course of the technological process and allows manufacturing high-quality products, while the selected strains can impart new, desired features to the products, including, in particular new aromas. It was found that KVEIK yeast fermentation performance was slightly slower compared to the popular US-05 strain producing slightly less alcohol and consuming lower amount of the extract. On the other hand, the utilization of dextrins was better in the case of two KVEIK strains compared to US-05. Therefore, the results of the study failed to point out the exact fermentation performance, which is rather strain-dependent. However, the analysis of volatile compounds produced, as well as the sensory evaluation of beers fermented by KVEIK yeasts, suggests that the finished products are far more complex in the taste and aroma than the beers fermented with the standard strain. Therefore, the application of KVEIK might be a promising option to expand the portfolio of beers produced within the “beer specialties” segment of the beer market, being one of the most dynamically growing segments in the Polish beer market. Since the study was performed in small-scale laboratory conditions, further research should focus on the fermentation performance and quality of beers fermented by KVEIK yeast on larger scales where important technological features, such as fermentation course and cell viability under high hydrostatic pressure, as well as the consistency of fermentation course and beer quality during several re-pitching cycles, would be evaluated to assess their suitability for large-scale beer production. It is also necessary to point out that that the commercially available KVEIK yeasts (such as those used in this study) might be composed of more than one strain of *S. cerevisiae*. Therefore, the fermentation results might vary from brewery to brewery or even from batch to batch as a consequence of variations of the processing conditions of the wort composition. Thus, in order to provide the potential of KVEIK yeasts in commercial beer production, their fermentation performance should be studied based on different fermentation conditions and evaluation of their effect on beer features.

## 3. Materials and Methods

### 3.1. Materials

#### 3.1.1. Raw Material

A pale ale malt (Viking Malt, Strzegom, Poland) was prepared from two-row malting barley. Its color is in the range of 4–7 EBC (European Brewery Convention) units. It imparts to the wort a golden color and a deep malt flavor. It is often used together with Pilsner malt to add a deeper malt flavor and enhance the color of the beer. It is used to produce all kinds of pale ale, bitter, and amber, and is also suitable for subtle color correction of classic lagers.

A Munich light malt (Viking Malt, Poland) light was prepared from two-row malting barley. Its color is in the range of 14–18 ECB. It brings amber color and aroma to beer. Drying temperatures in the range of 110–120 °C give the malt an aromatic, nutty character. High temperature results in significantly reduced enzyme activity compared to Pilsner malt. Munich malt is used to accentuate the malt and full-bodied flavor.

Marynka hops belong to the bitter hops. The alpha-acid content is 6.5%. They are characterized by a versatile aroma ranging from citrus and floral notes to herbal and spicy notes, as well as a high content of bitter substances. They are the most widely cultivated variety of hops in Poland and are used as T90 pellets.

Citra hops are classified as aromatic hops. The content of alpha-acid is at the level of 12%; therefore they can also be successfully used as bitter hops. They have citrus, tropical fruit, lime, mango, passion fruit, and pineapple aromas. They are mainly grown in the United States and are commonly used for “cold” hopping during silent fermentation. They are also used as T90 pellets.

Amarillo hops belong to the universal hops and are characterized by the content of alpha acids at the level of 8% and are used in beer to both develop bitterness and specific aromas at the end of brewing. They impart notes of orange, flowers, tropical fruit, peach, apricot, and grapefruit aromas. Grown in the USA, they are also used as T90 pellets.

#### 3.1.2. Biological Material

Hornindal Var KVEIK yeast (HVK), Hornindal, is a KVEIK variety shared with the world by Terje Raftevold from Hornindal, Norway. It produces an intense tropical flavor and aroma with notes of fresh mandarin, mango, and pineapple, and is perfect for fruit hops. The yeast was used from their own collection located on the agar slant. The optimal temperature of the fermentation process is in the range of 22–37 °C. Lida KVEIK yeast (LK) is from Grodås. It has a delicate, fruity profile with hints of milk and caramel. The temperature of the wort to which the yeast is infused should be approx. 33 °C. The yeast was used from their own collection located on the agar slant. Var KVEIK 2 yeast (VK2) is Norwegian yeast used from their own collection located on the agar slant. FM 53 Voss KVEIK yeast (FM53) is a *Saccharomyces cerevisiae* strain from Voss (purchased at Fermentum Mobile, Poland), courtesy of Sigmund Gjernes. It is characterized by fruity aromas and is recommended especially for Norwegian-style beers. It is a top fermenting yeast and the fermentation temperature range is 20–40 °C. SafAle US-05 yeast (US-05) is a *Saccharomyces cerevisiae* strain (Fermentis, Marq-en-Baroeul, France). It is an American top-fermenting yeast for the production of a balanced, dry beer with a clean profile. It is also characterized by a low level of diacetyl produced and medium flocculation. The temperature of the fermentation process is in the range of 18–28 °C. HVK and LK strains were obtained from White Labs (San Diego, CA, USA) and are commercially available under the numbers WLP521 and WLP4052, respectively. According to the producer’s information (LK White Labs (Chicago, IL, USA)), it is a monoculture isolated from the original KVEIK ring while the producer does not provide information about the composition of HVK except for the place of origin. VK2 originated from Omega Yeast (USA) and it is sold under the number OYL-071; the producer does not provide information about the strain homogeneity. The producer of FM53 Fermentum Mobile (Gdańsk, Poland) also does not provide the information about the yeast strain composition.

### 3.2. Methods

#### 3.2.1. Preparation of Biological Material

A wort with a 7 °Plato extract, comprising only malt pale ale, was used to propagate the inoculum. It was sterilized in laboratory conditions, cooled, and then, maintaining sterility, collected into prepared tubes and flasks. The biological material on the slants was transferred with a loop to test tubes containing 5 mL of wort. Dry yeast was rehydrated in saline and, similarly to the liquid yeast, was added with a pipette (0.5 mL) to the test tubes containing the wort. Then, the whole tubes were shaken for 24 h (250 rpm). Afterward, the propagated biological material was transferred into 100 mL Erlenmeyer flasks containing 50 mL of the wort and shaken for 24 h (250 rpm). In the next step, the content of 100 mL flasks was transferred to 1000 mL flasks with 500 mL of wort, and the yeast was incubated on magnetic stirrers at 800 rpm for 24 h. The entire propagation process was carried out at 30 °C. The obtained inoculum was used for the fermentation of the brewing wort.

#### 3.2.2. Preparation of Wort

A mash tun with a volume of 40 L was used to produce 25 L of wort. In the first step, 10 L of water were poured into a mash tun and heated with oat and wheat flakes to 100 °C in order to gelatinize the starch. Then, 11 L of water were added to reduce the temperature inside a mash tun to 68 °C. After cooling, pale ale and light Munich malts were added. The malt mashing process was run at this temperature for 1 h under periodical manual mixing. The effects of the mashing process were checked using the iodine test to control the starch content in the mash. The iodine test was negative. After the time had elapsed, the temperature was then gradually increased to 78 °C, in which the mash was kept for 10 min in order to deactivate the saccharification enzymes. In the next step, the mash was transferred to a previously prepared filtering ladle equipped with a steel braid and a drain tap, whereafter the bed had been deposited and sparged. For this stage, 18 L of preheated water with a temperature of 76 °C was used. Then, about 30 L of wort were taken and cooked in a 40 L brew kettle. When the wort was boiling, 40 g of Marynka hops were added. After this operation, the wort was boiled for 50 min, and 30 g of Citra hops were added, and after another 5 min, 20 g of Amarillo hops were added, and the mixture was boiled for another 5 min. Then, the wort was cooled to 30 °C using a stainless-steel immersion cooler. The wort prepared in this way with the extract of 11.5 °Plato was divided into five lots of 5 L, each with three replicates. Each wort was inoculated with 500 mL of yeast inoculum, being either SafAle US-05 yeast (US-05), Hornindal Var KVEIK, Lida KVEIK, Var KVEIK 2, or FM 53 Voss KVEIK. The samples were transferred to the temperature of 25 °C, where the primary fermentation took place. After 10 days, the samples were poured over the yeast sediment for secondary fermentation. The process was carried out for 14 days at the temperature of 25 °C. Three days before the end of this stage, cold hops were added to the fermentation tubes. Then, 0.6 g/L of Citra and Amarillo hops were added to produce beer. In the next stage, the hops were filtered off and the beer was poured into 0.5 L bottles. At the end, 7 g glucose/L of the beer were added and the product was aged for 21 days.

#### 3.2.3. Basic Physicochemical Parameters

Degree of fermentation, extract content, energy value, and density of beer, as well as concentration of ethyl alcohol, were measured with near-infrared (NIR) spectroscopy using an Anton Paar Alex 500 oscillating densitometer (Anton Paar, Graz, Austria). Beers were degassed, centrifuged (2675 centrifugal force (g), 6000 rpm, 10 min), filtered with diatomaceous earth (1 g/100 mL beer) on laboratory filter papers, and subjected to analyses. The pH value of beer was measured with a Mettler Toledo MP 240 pH-meter (Columbus, OH, USA). Analyses were carried out in three replications.

#### 3.2.4. High-Performance Liquid Chromatography (HPLC)

HPLC was employed to analyze the carbohydrate profile (dextrins, maltotriose, maltose, glucose), and contents of glycerol, as well as lactic and acetic acids. Degassed and centrifuged (2675 centrifugal force (RCF), 6000 rpm, 10 min) samples were diluted two times with redistilled water and filtered through nylon filters (mesh size of 0.22 µm) to chromatographic vials. Beer samples were analyzed using a Prominence liquid chromatograph (Shimadzu, Kyoto, Japan) equipped with a Rezex ROA-Organic Acid H^+^ column (300 × 4.6 mm; Phenomenex, Torrance, CA, USA). Measurement parameters were as follows: injection volume 20 μL, elution temperature 60 °C, flow rate 0.6 mL/min, mobile phase 0.005 M H_2_SO_4_, and thermostat refractometric detector at 50 °C. Concentrations of carbohydrates, organic acids, and glycerol were determined based on a five-point calibration curve integrated in Chromax 10.0 software (Pol-Lab, Warsaw, Poland). Analyses were carried out in three technical replications.

#### 3.2.5. Total Phenolics Content

Total phenolics content was determined with the spectrophotometric method based on the reaction with the Folin–Ciocalteu (F–C) reagent [28]. A diluted beer sample and F–C reagent were mixed in a cuvette and incubated for 3 min; afterwards, a 20% Na_2_CO_3_ solution and redistilled water were added to the mixture. The samples were then incubated in the dark for 60 min, and afterwards their absorbance was measured at a wavelength of 765 nm. Results were expressed as gallic acid equivalents (GAE) per 100 mL of beer. Analyses were carried out in three technical replications.

#### 3.2.6. Antioxidative Activity Assayed Based on the Test with DPPH^•^ Reagent

A diluted beer sample was mixed in a cuvette with DPPH^•^ dissolved in ethanol and water. The mixture was incubated at a room temperature for 10 min and, afterward, its absorbance was measured at a wavelength of 517 nm [29]. Results were expressed as Trolox equivalents (TE) per 1 L of beer (mmol TE/L). Analyses were carried out in three technical replications.

#### 3.2.7. Antiradical Activity Assayed Based on the Reaction with ABTS^•+^

A diluted beer sample was mixed in a cuvette with an ABTS^•+^ solution whose absorbance measured at a wavelength of 734 nm reached 0.700 [30]. Sample absorbance was measured after a six-minute incubation. Results were expressed as Trolox equivalents per 1 L of beer (mmTE/L). Analyses were carried out in three technical replications.

#### 3.2.8. Antioxidative Activity Assayed Based on the FRAP Test

A total of 0.2 mL of once-diluted beer sample was mixed in a cuvette with 3 mL of ferric complex (10 mmol 2,4,6-Tris(2-pyridyl)-s-triazine (TPTZ)/L reagent with 20 mmol/L ferric chloride in acetate buffer, pH 3.6) and 2 mL of redistilled water. After a 10-min incubation, absorbance was measured at a wavelength of 593 nm [31]. Results were expressed as Trolox equivalents per 1 L of beer (mmol TE/L). Analyses were carried out in three technical replications.

#### 3.2.9. Volatile Compounds Analysis

Volatile compounds in the tested beers were analyzed by the gas chromatography technique coupled with flame ionizing detector (GC-FID). A GC2010 Plus gas chromatograph with a FID-2010 detector and a headspace autosampler (HS-20) (Shimadzu Corporation, Kyoto, Japan), equipped with a CP-WAX 57 CB column (50 m × 0.32 mm ID × 0.2 µm) (Agilent Technologies, Santa Clara, CA, USA) by the modified version of Kłosowski and Mikulski were used in the study [32]. Beer samples were degassed, mixed with diatomaceous earth (1g per 100 mL of beer), and filtered through the paper filter. After the filtration, 10 mL of beer were transferred to the 20 mL headspace vial. Each vial was conditioned in the headspace autosampler oven set at 40 °C and equilibrated for 20 min at a shaking level of 2 prior to the injection of the sample into the column. Volume of the volatiles transferred to the column was equal to 1 mL, pressurizing time was equal to 0.5 min, pressurizing equilibration time was equal to 0.1 min, load time was equal to 0.5 min, load equilibrium time was equal to 0.1 min, injection time was equal to 0.5 min, needle flush time was set to 0 min, and total GC cycle time was equal to 60 min. Injection mode was set to split (split ratio 10), and the GC temperature program was as follows: 40 °C, hold 3 min, increase to 80 °C at the rate 5 °C per min, hold 3 min, increase to 140 °C at the rate of 10 °C per min, hold 9 min, increase to 160 °C at the rate of 20 °C, hold 4 min (total program time 34 min). Starting pressure was set at 100 kPa, starting flow was set at 6.6 mL/min, starting column flow was set at 0.33 mL/min, starting linear velocity was set at 11.8 cm/s, and purge flow was set at 3 mL/min. The carrier gas was helium. The FID operated at 280 °C at a sampling rate of 40 ms with the stop time at 34 min. The H_2_ flow to the FID was equal to 50 mL/min, air flow was equal to 400 mL/min, and makeup gas (helium) flow was equal to 30 mL/min. Data were integrated and quantitated in the LabSolutions software (Shimadzu Corporation, Kyoto, Japan). Automatic integration was performed with the following conditions: peak width equal to 3 s, slope at least 1000 uV/min, min. area 1000 counts. Identification of the compounds was performed using analytical standards, with identification method basing on absolute retention time. Quantitation was performed using external standards, with five calibration points (coefficient of determination R² was equal to at least 0.999).

#### 3.2.10. Sensory Analysis

The beers were subjected to a sensory evaluation. A group of 15 panelists consisting of M.Sc. and Ph.D. students from the Wrocław University of Environmental and Life Sciences, Wrocław, Poland, participated in the study. All panelists were educated by academic personnel who have experience in the field of beer sensory analysis. The group consisted of nine men and six women aged 24–28 years; 11 of the panelists had a bachelor’s level of education and 4 of them had a master’s degree (all of the panelists were educated in Food Technology and Human Nutrition with a specialization in fermentation technology). The panelists reported their beer preferences as “open to new types of beers”, “prefer ale beers to lagers”, “interested in new unconventional styles”, etc. The following beer quality attributes were assessed: foaminess, clarity, color, saturation, bitterness, aroma, and taste. The evaluation was made on a 2–5 point scale. Additionally, on a separate evaluation sheet created following the recommendations of the Polish Association of Home Brewers (PSPD), the aroma was assessed, divided into malt, hops, esters, or other aromas. The bitterness was assessed by dividing into intensity, quality, and finish. The taste of the beers was also assessed. During the study, the evaluating group could also write down their subjective sensory notes and detected defects of the evaluated beers. The analysis was carried out in a sensory analysis laboratory equipped with specially prepared stands.

#### 3.2.11. Statistics

Selected data were processed using Statistica 13.5 software (StatSoft, Tulsa, OK, USA), based on ANOVA (α = 0.05). Duncan test was used to analyze differences between mean results (*p* < 0.05).

## Figures and Tables

**Table 1:** Basic physicochemical parameters of beers at different production stages.

Yeast Culture	Stage of Brewing	Alcohol		Real Extract	Apparent Extract	RDF ^3^	ADF ^4^	Density	Energy Value	pH
		% *v*/*v*	% *w*/*w*	% *w*/*w*	% *w*/*w*	%	%	g/cm ³	kcal/100 mL	
	Wort	nd	nd ^2^	10.97 ± 0.03 ^a^	10.85 ± 0.00 ^a^	nd	nd	1.04198 ± 0.00 ^a^	36.34 ± 0.03 ^a,b,c^	6.16 ± 0.00 ^a^
US-05	After primary fermentation	3.97 ± 0.04 ^e,1^	3.14 ± 0.01 ^d^	4.64 ± 0.15 ^b,c^	2.94 ± 0.14 ^bc^	56.61 ± 0.15 ^g^	73.10 ± 0.77 ^c,d^	1.01005 ± 0.01 ^a^	37.53 ± 0.59 ^a,b^	5.00 ± 0.17 ^a^
HVK		3.50 ± 0.11 ^f^	2.71 ± 0.13 ^e,f^	4.56 ± 0.06 ^b,c^	2.95 ± 0.06 ^bc^	58.23 ± 0.01 ^e,f,g^	72.03 ± 0.37 ^c,d^	1.01005 ± 0.01 ^a^	34.32 ± 0.97 ^b,c,d^	4.53 ± 0.28 ^b,c^
LK		3.76 ± 0.20 ^e,f^	2.96 ± 0.18 ^d,e^	4.50 ± 0.11 ^b,c^	2.98 ± 0.08 ^bc^	58.27 ± 0.31 ^d,e,f,g^	71.85 ± 0.22 ^c,d^	1.01007 ± 0.01 ^a^	36.30 ± 1.53 ^a,b,c^	4.85 ± 0.01 ^a,b^
VK2		3.76 ± 0.17 ^e,f^	2.94 ± 0.08 ^d,e,f^	4.71 ± 0.04 ^b^	3.07 ± 0.01 ^bc^	57.97 ± 0.18 ^f,g^	70.18 ± 0.18 ^d^	1.01005 ± 0.00 ^a^	36.23 ± 1.15 ^a,b,c^	4.60 ± 0.05 ^a,b,c^
FM53		3.40 ± 0.23 ^f^	2.64 ± 0.23 ^f^	4.83 ± 0.04 ^b^	3.17 ± 0.02 ^b^	56.61 ± 0.15 ^g^	73.10 ± 0.77 ^c,d^	1.01115 ± 0.01 ^a^	34.37 ± 2.11 ^b,c,d^	4.70 ± 0.38 ^a,b,c^
US-05	After secondary fermentation	4.83 ± 0.11 ^c,d^	3.78 ± 0.10 ^a,b,c^	3.96 ± 0.22 ^c,d^	2.61 ± 0.24 ^c^	64.17 ± 1.49 ^b^	82.63 ± 2.62 ^b^	1.00960 ± 0.01 ^a^	37.48 ± 0.57 ^a,b^	4.56 ± 0.06 ^b,c^
HVK		4.65 ± 0.11 ^d^	3.64 ± 0.09 ^c^	4.66 ± 0.01 ^b,c^	3.09 ± 0.03 ^bc^	60.21 ± 0.49 ^c,d^	74.61 ± 0.60 ^c^	1.00980 ± 0.01 ^a^	33.86 ± 1.30 ^c,d^	4.40 ± 0.03 ^c^
LK		4.70 ± 0.08 ^d^	3.68 ± 0.08 ^a,b,c^	4.23 ± 0.30 ^b,c^	3.05 ± 0.10 ^bc^	61.86 ± 0.95 ^c^	79.87 ± 1.85 ^b^	1.00956 ± 0.01 ^a^	33.10 ± 2.72 ^c,d^	4.28 ± 0.04 ^c^
VK2		4.71 ± 0.01 ^d^	3.68 ± 0.01 ^a,b,c^	4.74 ± 0.06 ^b^	3.16 ± 0.03 ^b^	60.07 ± 0.26 ^c,d,e^	74.43 ± 0.32 ^c^	1.01005 ± 0.01 ^a^	33.24 ± 0.71 ^c,d^	4.39 ± 0.03 ^c^
FM53		4.65 ± 0.02 ^d^	3.63 ± 0.01 ^c^	4.74 ± 0.02 ^b^	3.19 ± 0.01 ^b^	59.81 ± 0.00 ^d,e,f^	74.00 ± 0.00 ^c^	1.01009 ± 0.01 ^a^	31.35 ± 0.47 ^d^	4.52 ± 0.04 ^b,c^
US-05	After aging	5.58 ± 0.29 ^a^	3.97 ± 0.30 ^a,b^	2.98 ± 0.67 ^e^	0.95 ± 0.13 ^d^	70.15 ± 0.14 ^a^	89.26 ± 3.71 ^a^	1.00208 ± 0.01 ^b^	38.03 ± 0.83 ^a^	4.83 ± 0.25 ^a,b^
HVK		5.23 ± 0.29 ^a,b,c^	3.99 ± 0.04 ^a^	3.16 ± 0.46 ^e^	1.26 ± 0.43 ^de^	71.09 ± 0.80 ^a^	88.61 ± 1.39 ^a^	1.00306 ± 0.01 ^b^	35.16 ± 1.63 ^a,b,c^	4.64 ± 0.17 ^a,b,c^
LK		5.36 ± 0.35 ^a,b^	3.94 ± 0.13 ^a,b,c,d^	3.12 ± 0.29 ^e^	1.20 ± 0.23 ^de^	71.28 ± 1.17 ^a^	89.06 ± 2.17 ^a^	1.00286 ± 0.01 ^b^	35.39 ± 2.03 ^a,b,c^	4.56 ± 0.21 ^b,c^
VK2		5.28 ± 0.22 ^a,b^	3.73 ± 0.03 ^a,b,c^	3.43 ± 0.49 ^d,e^	1.50 ± 0.23 ^d^	70.24 ± 2.01 ^a^	88.45 ± 1.00 ^a^	1.00403 ± 0.01 ^b^	35.92 ± 0.66 ^a,b,c^	4.58 ± 0.13 ^b,c^
FM53		5.05 ± 0.22 ^b,c,d^	3.66 ± 0.17 ^b,c^	3.23 ± 0.37 ^e^	1.55 ± 0.46 ^d^	69.79 ± 0.46 ^a^	86.99 ± 2.68 ^a^	1.00420 ± 0.01 ^b^	34.04 ± 0.10 ^b,c,d^	4.70 ± 0.13 ^a,b,c^

^1^ Values are expressed as the mean (*n* = 3) ± standard deviation. Mean values with different letters (a, b, c, etc.) within the same row are statistically different (*p*-value < 0.05); ^2^ nd, not detected; ^3^ RDF, real degree of fermentation; ^4^ ADF, apparent degree of fermentation.

**Table 2:** Concentrations of starch hydrolysis products (glucose, maltose, maltotriose, and dextrins) and glycerol in the beers.

Yeast Culture	Stage of Brewing	Dextrins	Maltotriose	Maltose	Glucose	Glycerol	Lactic Acid	Acetic Acid
		g/L						
	Wort	31.53 ± 0.79 ^a,1^	13.62 ± 0.32 ^a^	47.34 ± 0.78 ^a^	6.47 ± 0.12 ^a^	nd ^2^	nd	nd
US-05	After primary fermentation	29.27 ± 0.37 ^b^	2.01 ± 0.12 ^b^	nd	0.66 ± 0.28 ^c^	1.29 ± 0.03 ^c^	nd	nd
HVK		29.56 ± 0.45 ^b^	1.19 ± 0.27 ^c^	nd	nd	1.48 ± 0.03 ^a^	nd	nd
LK		29.28 ± 0.69 ^b^	nd	nd	2.26 ± 0.39 ^b^	1.42 ± 0.08 ^b^	nd	nd
VK2		31.03 ± 0.19 ^a^	nd	nd	nd	1.46 ± 0.01 ^a,b^	nd	nd
FM53		29.29 ± 0.39 ^b^	1.38 ± 0.13 ^c^	nd	nd	1.51 ± 0.01 ^a^	nd	nd
US-05	After secondary fermentation	21.04 ± 0.02 ^c^	nd	nd	0.48 ± 0.06 ^c^	1.24 ± 0.08 ^b^	nd	nd
HVK		27.97 ± 0.31 ^a^	nd	nd	nd	1.49 ± 0.03 ^a^	nd	nd
LK		16.35 ± 0.15 ^d^	nd	nd	nd	0.99 ± 0.01 ^c^	nd	nd
VK2		26.57 ± 0.46 ^b^	nd	nd	nd	1.26 ± 0.04 ^b^	nd	nd
FM53		28.84 ± 0.43 ^a^	nd	nd	nd	1.48 ± 0.01 ^a^	nd	nd
US-05	After aging	13.51 ± 0.56 ^d^	nd	nd	nd	1.31 ± 0.11 ^c^	nd	nd
HVK		23.97 ± 0.05 ^a^	nd	nd	nd	1.29 ± 0.01 ^c^	nd	nd
LK		14.07 ± 0.01 ^b^	nd	nd	nd	1.47 ± 0.01 ^b^	nd	nd
VK2		18.67 ± 0.15 ^b^	nd	nd	nd	1.51 ± 0.03 ^a,b^	nd	nd
FM53		16.66 ± 0.18 ^c^	nd	nd	nd	1.63 ± 0.04 ^a^	nd	nd

^1^ Values are expressed as the mean (*n* = 3) ± standard deviation. Mean values with different letters (a, b, c, etc.) within the same row are statistically different (*p*-value < 0.05); ^2^ nd, not detected.

**Table 3:** Total phenolics content (TPC) and antioxidative activity of the beers.

Yeast Culture	Stage of Brewing	TPC	DPPH	ABTS^•+^	FRAP
		mg GAE/100 mL	mM TE/mL		
	Wort	16.01 ± 0.70 ^e,1^	0.81 ± 0.04 ^e^	4.54 ± 0.48 ^d^	0.37 ± 0.05 ^d^
US-05	After primary fermentation	33.40 ± 0.51 ^a^	1.42 ± 0.06 ^c^	5.89 ± 0.26 ^c^	0.95 ± 0.14 ^b^
HVK		27.93 ± 0.77 ^c^	1.30 ± 0.04 ^d^	6.18 ± 0.23 ^b,c^	0.82 ± 0.05 ^b^
LK		32.00 ± 1.05 ^a,b^	2.07 ± 0.03 ^a^	6.42 ± 0.20 ^b^	0.96 ± 0.07 ^b^
VK2		30.67 ± 1.59 ^b^	1.16 ± 0.04 ^e^	5.97 ± 0.20 ^b,c^	1.22 ± 0.05 ^a^
FM53		21.97 ± 0.94 ^d^	1.57 ± 0.04 ^b^	6.92 ± 0.36 ^a,b^	0.39 ± 0.16 ^c^
US-05	After secondary fermentation	35.43 ± 0.88 ^a^	1.14 ± 0.80 ^b^	5.29 ± 0.62 ^c^	1.06 ± 0.08 ^a^
HVK		25.31 ± 0.52 ^d^	1.89 ± 0.04 ^a^	6.66 ± 0.28 ^a,b^	0.48 ± 0.15 ^c^
LK		25.85 ± 0.74 ^d^	1.63 ± 0.05 ^a,b^	7.18 ± 0.65 ^a,b^	0.75 ± 0.22 ^b^
VK2		29.52 ± 1.07 ^b^	1.44 ± 0.05 ^a,b^	7.45 ± 0.23 ^a^	0.83 ± 0.11 ^a,b^
FM53		27.44 ± 0.44 ^c^	1.14 ± 0.80 ^b^	6.45 ± 0.26 ^b^	0.98 ± 0.08 ^a,b^
US-05	After aging	29.64 ± 0.84 ^b^	1.90 ± 0.07 ^b^	5.71 ± 0.59 ^c^	1.33 ± 0.08 ^a^
HVK		27.53 ± 0.95 ^c^	1.17 ± 0.06 ^e^	6.78 ± 0.14 ^a,b^	0.92 ± 0.08 ^c^
LK		17.97 ± 1.17 ^d^	2.00 ± 0.04 ^a^	7.21 ± 0.30 ^a^	0.91 ± 0.06 ^c^
VK2		27.39 ± 1.38 ^c^	1.53 ± 0.04 ^c^	6.52 ± 0.35 ^b^	0.94 ± 0.12 ^c^
FM53		33.26 ± 0.69 ^a^	1.41 ± 0.03 ^d^	6.78 ± 0.20 ^a,b^	1.14 ± 0.13 ^b^

^1^ Values are expressed as the mean (*n* = 3) ± standard deviation. Mean values with different letters (a, b, c, etc.) within the same row are statistically different (*p*-value < 0.05).

**Table 4:** Analysis of volatile compounds of the beers.

Volatile Compounds	Chemical Family	Beer				
		US-05	HVK	LK	VK2	FM-53
		[mg/L]				
Acetaldehyde	Aldehydes	4.05 ± 0.07 ^b,1^	1.83 ± 0.26 ^d^	5.10 ± 0.19 ^a^	5.25 ± 0.24 ^a^	3.60 ± 0.07 ^b,c^
Propanal	Aldehydes	0.73 ± 0.18 ^a^	0.46 ± 0.02 ^a,b,c^	0.43 ± 0.06 ^a,b,c^	0.64 ± 0.04 ^a,b^	0,69 ± 0.01 ^a^
Hexanal	Aldehydes	0.067 ± 0.00 ^a^	0.046 ± 0.007 ^d^	0.054 ± 0.00 ^c^	0.062 ± 0.00 ^a,b^	0.056 ± 0.00 ^b,c^
Furfural	Aldehydes	9.19 ± 0.41 ^a^	8.47 ± 0.54 ^a,b^	6.57 ± 0.68 ^b^	5.65 ± 0.19 ^b^	7.48 ± 0.24 ^a,b^
2-butanol	Alcohols	0.55 ± 0.01 ^a^	nd	0.52 ± 0.02 ^a^	0.44 ± 0.01 ^a,b^	0.17 ± 0.1 ^b,c^
1-propanol	Alcohols	29.48 ± 1.43 ^b,c,d^	33.43 ± 0.01 ^b^	32.70 ± 1.30 ^b,c^	70.22 ± 1.91 ^a^	28.70 ± 1.41 ^c,d^
Isobutanol	Alcohols	38.56 ± 1.36 ^c^	42.58 ± 0.41 ^b^	40.25 ± 0.86 ^b,c^	56.97 ± 1.06 ^a^	38.41 ± 1.36 ^c^
1-hexanol	Alcohols	0.03 ± 0.02 ^a,b,c^	0.03 ± 0.02 ^a,b,c^	0.03 ± 0.01 ^a,b,c^	0.05 ± 0.01 ^a^	0.01 ± 0.01 ^c^
2-pentanol	Alcohols	nd ^2^	trace	0.14 ± 0.05 ^b^	nd	0.49 ± 0.16 ^a^
1-butanol	Alcohols	0.50 ± 0.02 ^b^	0.53 ± 0.01 ^b^	0.61 ± 0.03 ^a^	0.25 ± 0.02 ^c^	0.52 ± 0.01 ^b^
2-methylbutanol	Alcohols	33.15 ± 1.16 ^b^	35.38 ± 0.61 ^a^	32.89 ± 0.98 ^b^	28.09 ± 0.58 ^c^	32.36 ± 1.21 ^b^
3-methylbutanol	Alcohols	115.22 ± 4.81 ^b^	124.70 ± 0.95 ^a^	118.13 ± 2.40 ^b^	86.59 ± 1.87 ^c^	122.77 ± 4.47 ^a^
Phenylethyl alcohol	Alcohols	36.59 ± 3.33 ^b,c^	39.20 ± 3.10 ^b^	31.40 ± 5.99 ^b,c^	29.27 ± 1.32 ^b,c^	55.68 ± 2.31 ^a^
2,5-dimethylpyrazine	Pyrazines	1.11 ± 0.11 ^a,b^	0.80 ± 0.06 ^c^	0.93 ± 0.11 ^b,c^	1.18 ± 0.01 ^a,b^	1.21 ± 0.02 ^a^
2,3-dimethylpyrazine	Pyrazines	0.06 ± 0.04 ^a^	0.12 ± 0.02 ^a^	trace	0.04 ± 0.02 ^a^	0.07 ± 0.04 ^a^
2,3-diethylpyrazine	Pyrazines	0.43 ± 0.02 ^a,b^	0.42 ± 0.01 ^b^	0.45 ± 0.01 ^a^	0.43 ± 0.01 ^a,b^	nd
Ethyl octanoate	Esters	0.80 ± 0.02 ^c^	0.90 ± 0.03 ^b^	0.95 ± 0.02 ^a,b^	1.00 ± 0.03 ^a^	1.01 ± 0.01 ^a^
Ethyl acetate	Esters	120.86 ± 0.29 ^a^	96.72 ± 1.56 ^c^	114.54 ± 1.10 ^b^	96.59 ± 2.59 ^c^	95.95 ± 0.58 ^c^
Isopentyl acetate	Esters	0.67 ± 0.01 ^a^	0.55 ± 0.02 ^d^	0.64 ± 0.02 ^b^	0.51 ± 0.01 ^e^	0.61 ± 0.01 ^c^
Ethyl decanoate	Esters	3.31 ± 0.61 ^d^	9.87 ± 0.30 ^b^	12.16 ± 0.38 ^a^	7.26 ± 0.38 ^c^	10.62 ± 0.33 ^b^
Ethyl hexanoate	Esters	0.38 ± 0.01 ^b^	0.30 ± 0.01 ^c^	0.35 ± 0.02 ^b,c^	0.35 ± 0.02 ^b,c^	0.44 ± 0.02 ^a^
Total volatiles		395.74	396.34	398.84	390.84	400.846

^1^ Values are expressed as the mean (*n* = 3) ± standard deviation. Mean values with different letters (a, b, c, etc.) within the same row are statistically different (*p*-value < 0.05); ^2^ nd, not detected.

**Table 5:** Results of the sensory analysis of the beers.

Yeast Culture	Foaminess	Clarity	Color	Saturation	Bitterness	Aroma	Flavor	Total Points
US-05	8.13 ± 1.60 ^a,b,1^	3.60 ± 1.06 ^a^	4.27 ± 0.88 ^a^	3.77 ± 1.03 ^a^	13.60 ± 4.22 ^a^	14.13 ± 4.23 ^b,c^	21.00 ± 7.94 ^c^	68.47
HVK	8.13 ± 1.19 ^a,b^	4.00 ± 0.93 ^a^	4.53 ± 0.74 ^a^	4.00 ± 0.65 ^a^	14.40 ± 4.22 ^a^	17.07 ± 2.81 ^a^	26.60 ± 5.42 ^a,b^	78.73
LK	7.47 ± 1.6 ^b^	3.93 ± 0.80 ^a^	4.47 ± 0.83 ^a^	4.27 ± 0.88 ^a^	15.20 ± 3.10 ^a^	15.73 ± 3.84 ^a,b,c^	27.07 ± 5.84 ^a,b^	78.13
VK2	8.76 ± 1.23 ^a^	3.93 ± 0.88 ^a^	4.07 ± 0.887 ^a^	3.87 ± 0.92 ^a^	13.33 ± 3.60 ^a^	13.60 ± 3.94 ^c^	22.80 ± 8.56 ^b,c^	70.33
FM53	8.27 ± 1.28 ^a,b^	4.13 ± 0.74 ^a^	4.13 ± 0.92 ^a^	4.07 ± 0.96 ^a^	14.13 ± 3.66 ^a^	16.53 ± 2.56 ^a,b^	29.87 ± 6.19 ^a^	81.13

^1^ Values are expressed as the mean ± standard deviation. Mean values with different letters (a, b, c) within the same row are statistically different (*p*-value < 0.05).

## Data Availability

Not applicable.

## Sample Availability

Samples of the analyzed compounds are available from the authors.
